# *Cutibacterium acnes* in spine tissue: characteristics and outcomes of non-hardware-associated vertebral osteomyelitis

**DOI:** 10.5194/jbji-8-143-2023

**Published:** 2023-04-25

**Authors:** Matteo Passerini, Julian Maamari, Don Bambino Geno Tai, Robin Patel, Aaron J. Tande, Zelalem Temesgen, Elie F. Berbari

**Affiliations:** 1 Division of Public Health, Infectious Diseases, and Occupational Medicine, Mayo Clinic, Rochester, MN, USA; 2 Division of Infectious Diseases and International Medicine, Department of Medicine, University of Minnesota, Minneapolis, MN, USA; 3 Division of Clinical Microbiology, Department of Laboratory Medicine and Pathology, Mayo Clinic, Rochester, MN, USA

## Abstract

*Cutibacterium acnes* isolation from spine tissue can be challenging because the organism can represent a contaminant. There is a paucity of data regarding the role of *C. acnes* in non-hardware-associated vertebral osteomyelitis (VO). Herein we evaluate the clinical and microbiological characteristics, treatment, and outcome of patients with *C. acnes* VO. Data were retrospectively collected from adults with a positive spine culture for *C. acnes* at Mayo Clinic, Rochester (MN), from 2011 to 2021. Patients with spinal hardware and polymicrobial infections were excluded. Of the subjects, 16 showed radiological and clinical findings of VO: 87.5 % were male, the average age was 58 years ( ±15 SD), and back pain was the predominant symptom. Of the lesions, 89.5 % involved the thoracic spine. Of the subjects, 69 % had experienced an antecedent event at the site of VO. In five subjects, *C. acnes* was isolated after 7 d of anaerobic culture incubation. Thirteen subjects were treated with parenteral β -lactams, and three with oral antimicrobials, without any evidence of recurrence. Twenty-one subjects were not treated for VO, as *C. acnes* was considered a contaminant; at follow-up, none had evidence of progressive disease. *C. acnes* should be part of microbiological differential diagnosis in patients with suspected VO, especially in the context of a prior spinal procedure. Anaerobic spine cultures should undergo prolonged incubation to enable recovery of *C. acnes*. *C. acnes* VO may be managed with oral or parenteral antimicrobial therapy. Without clinical and radiological evidence of VO, a single positive culture of *C. acnes* from spine tissue frequently represents contaminants.

## 1 Introduction

*Cutibacterium acnes* is an anaerobic, non-spore-forming, gram-positive rod. It primarily colonizes the sebaceous glands and hair follicles of human skin, but it has also been detected in the oral cavity and gastrointestinal and genitourinary tracts (Achermann et al., 2014; Grice et al., 2009). Although often considered a contaminant, it has been associated with a variety of infections (Kanafani, 2022), and, recently, potentially other diseases and auto-inflammatory disorders (Leheste et al., 2017; Zimmermann and Curtis, 2019). Infections due to *C. acnes* can be divided into (a) skin infections, such as acne, although its pathogenetic role is still under investigation (van Steensel and Goh, 2021); (b) surgical wound infections; and (c) deep-seated infections. Among the latter, the most common clinical manifestations are orthopedic implant-related infections, including those involving prosthetic joints (especially shoulder arthroplasties) and spine hardware, given the higher density of *C. acnes* in this area (MacLean et al., 2019; Zeina A Kanafani, 2022). It has also been associated with native vertebral osteomyelitis (VO) and other infections (Cobo et al., 2018). Some evidence suggests a potential role for *C. acnes* in degenerative spine conditions, such as degenerative disk disease, Modic changes, and disk herniation (Iyer et al., 2019; Khalil et al., 2019). There are a few case reports and small case series of *C. acnes* VO; some did not distinguish hardware-associated from non-hardware-associated cases (Beatty et al., 2019; Kowalski et al., 2007; Uçkay et al., 2010). Given the scarcity of data, its indolent nature, and the paucity of clinical signs and symptoms, distinguishing clinically insignificant cultures from an infection requiring treatment may be challenging. The objective of this study is to present the experience at a single center by describing the clinical presentation, microbiological features, treatment, and outcomes of patients with *C. acnes* VO, with a focus on the outcome of patients with clinically insignificant isolates who did not receive antimicrobial therapy.

## 2 Methods

### 2.1 Microbiology and species identification

Patients with positive vertebral bone cultures for *C. acnes* at Mayo Clinic, Rochester (MN), from January 2011 to December 2021 were identified through the laboratory information system. Cultures were incubated until positive or for a maximum time of 14 d. Species identification was determined using matrix-assisted laser desorption ionization time-of-flight mass spectrometry (Bruker, Bremen, Germany), with 16S ribosomal RNA gene sequencing utilized as needed.

### 2.2 Patients

Clinical, microbiological, radiographic, and follow-up data were retrospectively collected. Patients with *C. acnes* spinal hardware-associated infections or polymicrobial infection were excluded. Patients with isolation of other species of *Cutibacterium*, infections of the skull, surgical wound infections, age <18 years, no research authorization to use their data, or missing data to evaluate outcomes at the end of the treatment were also excluded. For patients with *C. acnes* VO, demographics (age, sex, body mass index, comorbidities), microbiological features, presence of a distant focus of infection, radiological features, treatment, and follow-up data were abstracted. Follow-up data for patients with clinically insignificant *C. acnes* were also collected.

### 2.3 Definitions

*C. acnes* VO was defined as the presence of (a) two spine cultures that were positive for *C. acnes*, and there was radiological and/or clinical suspicion of VO; or (b) one spine culture was positive for *C. acnes*, and there was radiological and/or clinical suspicion of VO followed by improvement of symptoms or imaging after treatment.

Clinically insignificant *C. acnes* was defined as isolation from one or more samples in the absence of radiological and/or clinical suspicion of VO or when an alternative diagnosis was present.

### 2.4 Statistical analysis

Frequency counts and percentages were used for categorical variables. Medians with interquartile ranges (IQRs) or ranges and means with standard deviation (SD) were used for continuous variables according to the distribution of the data. For comparison between the group of *C. acnes* VO and clinically insignificant *C. acnes*, Pearson's chi-squared test was used for categorical variables. In cases where the sample size was small, Fisher's exact test was used. Mann–Whitney test was performed for continuous variables. The study was deemed exempt by the Mayo Clinic Institutional Review Board.

**Figure 1:**
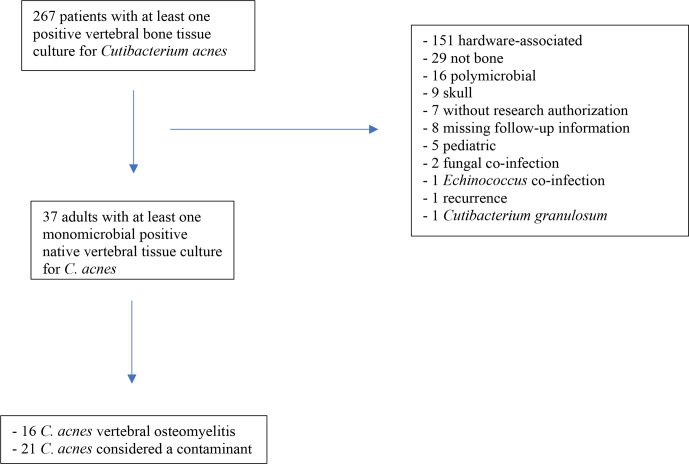
Cases of *Cutibacterium acnes* in spine tissue at Mayo Clinic between 2011–2021.

## 3 Results

There were 267 patients with at least one spine tissue culture positive for *C. acnes* during the study period. The main reasons for the exclusion of patients are outlined in Fig. 1. Of the patients,16 met the a priori *C. acnes* VO definition and 21 with clinically insignificant *C. acnes*. Clinical characteristics, treatment, and outcomes of all patients are summarized in Table 1.

### 3.1 *C. acnes* VO

Most patients with VO were male (88 %). The average age was 58 years (SD ±15 ). Most had comorbidities (63 %). Back pain was the predominant presenting symptom (100 %), while fever and neurological deficits were uncommon (one and three patients, respectively). Nine patients (56 %) presented with an elevated C-reactive protein, with four (25 %) having an elevated serum white blood cell count. One patient had concomitant *C. acnes* bloodstream infection without endocarditis. Half of the patients had epidural involvement, with four having an epidural or paraspinal abscess. Most detected lesions were localized in the thoracic spine, with no lumbar lesions. Most patients (69 %) had a history of a prior spine procedure or trauma. Seven had a previous surgical spinal intervention; among these, three had a previous vertebral decompression, two had a previous hemilaminectomy, one had a vertebroplasty, and one had a discectomy. Two patients had had spinal injections; among these, one had epidural injection for pain, and one had an intrathecal injection of stem cells. Two patients had experienced antecedent trauma at the site of VO. The median time from these events to the onset of symptoms was 80 d (IQR: 26–165). The diagnosis was made with CT-guided biopsy in 13 patients, with the remainder made with an open biopsy. Eight patients had two or more positive cultures for *C. acnes*. The median time to positivity was 5 d (range: 3–13 d). For five patients (31 %), the time to positivity was 7 or more days. Notably, 1/16 patients was receiving antimicrobial therapy at the time of microbiological diagnosis.

**Table 1:** Clinical, demographic, radiological, and microbiological characteristics of patients included in the study.

	*C. acnes* vertebral	*C. acnes* clinically	P value
	osteomyelitis ( n=16 )	insignificant ( n=21 )	
Male; n (%)	14 (88)	16 (76)	0.67
Age; average ( ± SD ${}^{a}$ )	58 (15)	67 (17)	0.07
BMI ${}^{b}$ ; median (IQR ${}^{c}$ )	28 (23–32)	28 (24–31)	0.91
Comorbidities; n (%)			
Diabetes mellitus	6 (38)	4 (19)	0.27
Immunosuppression	2 (13)	2 (10)	0.99
Dialysis	1 (6)	0 (0)	0.43
Person who injects drugs	1 (6)	0 (0)	0.43
Active malignancy	0 (0)	4 (19)	0.11
Risk factors for infection; n (%)			
Prior surgery	7 (44)	3 (14)	0.06
Prior injections	2 (13)	1 (5)	0.56
Prior trauma	2 (13)	0 (0)	0.188
Surgery and injections and trauma	11 (69)	4 (19)	0.006
Symptoms; n (%)			
Back pain	16 (100)	14 (67)	0.01
Fever	1 (6)	1 (5)	0.99
Neurological deficit	3 (19)	7 (33)	0.46
Inflammatory markers at presentation; n (%)			
Elevated blood leukocytes count ${}^{d}$	4 (25)	5 (24)	0.99
Elevated C-reactive protein ${}^{e}$	9 (56)	4 (19)	0.03
Bacteremia; n (%)	1 (6)	0 (0)	0.43
Endocarditis; n (%)	0 (0)	0 (0)	0.99
Suspicion before tissue cultures; n (%)			
Vertebral osteomyelitis	16 (100)	5 (24)	0.00001
Malignancy	0 (0)	14 (67)	0.0001
Other	0 (0)	2 (10)	0.49
Site of lesion; n (%)			
Cervical	2 (13)	2 (10)	0.99
Cervicothoracic	6 (38)	1 (5)	0.02
Thoracic	9 (64)	10 (48)	0.74
Thoracolumbar	2 (13)	1 (5)	0.56
Lumbar	0 (0)	5 (24)	0.05
Lumbosacral	0 (0)	0 (0)	0.99
Complications; n (%)			
Epidural involvement	8 (50)	3 (14)	0.03
Abscess	4 (25)	0 (0)	0.02
MRI ${}^{f}$ findings; n (%)			
Disk involvement ${}^{g}$	6 (43)	1 (10)	0.17
Vertebral bodies involvement ${}^{g}$	12 (86)	9 (90)	1
Soft tissue enhancement ${}^{g}$	8 (57)	2 (20)	0.1

**Table 1:** Continued.

	*C. acnes* vertebral	*C. acnes* clinically	P value
	osteomyelitis ( n=16 )	insignificant ( n=21 )	
Microbiological features; n (%)			
Two ( ≥ ) positive cultures	8 (50)	0 (0)	/
One positive culture	8 (50)	21 (100)	/
Time to first positivity; days (median, range)	5 (3–13)	8 (4–14)	0.007
Histology; n (%)			
Suggestive of inflammation	6 (38)	0 (0)	0.003
Not suggestive of inflammation	5 (31)	21 (100)	0.000001
Not performed	5 (31)	0 (0)	0.01
Treatment; n (%)			
Antibiotic alone	14 (88)	/	
Intravenous antibiotic therapy	13 (81)	/	
Oral antibiotic therapy	3 (19)	/	
Outcome			
Failure or relapse; n (%)	0 (0)	/	
Follow-up; weeks (median, IQR)	167 (71–305)	85 (4–488)	0.001

${}^{a}$ SD: standard deviation. ${}^{b}$ BMI: body mass index. ${}^{c}$ IQR: interquartile range. ${}^{d}$ Reference range: 3.4– $9.6\times 10^{9}$ L ${}^{-1}$ . ${}^{e}$ Reference range: ≤8.0 mg L ${}^{-1}$ . ${}^{f}$ MRI: magnetic resonance imaging. ${}^{g}$ MRI available for 14/16 *C.acnes* VO and for 10/21 *C.acnes* as clinically insignificant.

Most patients (88 %; n=14 ) were managed with antibiotic therapy only. Thirteen received parenteral β -lactams, of which 10 were treated with ceftriaxone. Seven of 13 patients received 6 weeks of therapy, with the others receiving a longer course. Three patients received oral therapy for 6 weeks: two patients received moxifloxacin and one doxycycline. Two patients developed *Clostridioides difficile*-associated infection during therapy, both while receiving ceftriaxone. Two patients were managed with spinal debridement in addition to antibiotic therapy. Treatment was successful in all the patients at the end of the therapy, with no relapse after a median follow-up of 167 weeks (IQR: 71–305).

### 3.2 Clinically insignificant *C. acnes*

Twenty-one patients with isolation of *C. acnes* from a single culture were not treated, and the microorganism was considered a contaminant. In these cases, there was no clinical or radiological suspicion of VO. The majority were male (76 %) with an average age of 67 years (SD ±17 ). The main reason for spine biopsy was suspicion of cancer due to radiological imaging and clinical presentation (76 %). Three patients underwent a biopsy because of indeterminate imaging: one underwent a surgical procedure to repair a dural leak, one was taken to the operating room urgently for cervical myelopathy caused by a degenerative disk collapse. The final diagnosis in 19 patients was a malignancy. The median time to culture positivity was 8 d (IQR: 6–11). After a median follow-up of 85.2 weeks (range: 4–488), none of these patients showed evidence of VO.

### 3.3 Comparison of the two groups

The main difference between the two groups of patients was the initial clinical and radiological suspicion. Five of 21 patients in the contaminant *C. acnes* group were suspected of having VO before results of tissue cultures and histopathology were available, which ultimately showed malignancy. Elevated C-reactive protein was significantly more common among patients with *C. acnes* VO than clinically insignificant *C. acnes* (64 % vs. 19 %; p=0.03 ). Compared to the group with *C. acnes* VO, the time to first culture positivity was longer in the group with *C. acnes* considered a contaminant (5 d with a range of 3–13 vs. 8 d with a range 4–14; p=0.007 ).

## 4 Discussion

In the absence of clinical, radiological, or histopathological signs of infection, a single culture yielding *C. acnes* from non-instrumented spine sites usually represent contamination. There were, however, patients with *C. acnes* VO, mainly in the context of a prior spine procedure or trauma. One-third of clinically significant *C. acnes* were recovered in culture after 1 week of anaerobic culture incubation; treatment success was 100 %.

*C. acnes* VO is a relatively rare entity (Abolnik et al., 1995; Harris et al., 2005; Hernández-Palazón et al., 2003; Kowalski et al., 2007), representing up to 4 % of bacterial VO (Carragee, 1997). Although some case series do not clearly distinguish between native and hardware-associated *C. acnes* infections (Uçkay et al., 2010), this distinction is crucial, as these represent two different entities. A previous case series described nine patients with *C. acnes* VO (Kowalski et al., 2007). The authors included patients with isolation of *C. acnes* from at least two specimens and excluded patients with hardware and mixed infections. Like the results presented here, the clinical presentation was generally mild, with only one patient having elevated inflammatory markers. Six of nine patients had had a previous spinal procedure. *C. acnes* should be part of the differential diagnosis of patients with suspected VO and a prior procedure (e.g., spinal surgery, spinal injections, trauma). Given the indolent nature of *C. acnes* infection, the prior intervention may be in a distant past, as shown here, where the median time from the previous spine events was 80 d. It is postulated that *C. acnes* may have been seeded into the site, formed a biofilm, and manifested clinically thereafter.

The results of this study provide intriguing information regarding the time to positivity of cultures. Five of 16 patients with VO and 14 of 25 with clinically insignificant *C. acnes* showed initial growth at or after 7 d of anaerobic incubation, reflecting the slow-growing nature of *C. acnes*. Incubation of anaerobic cultures of spinal specimens for 14 d is recommended to recover *C. acnes* in patients with suspected VO. The time to first positivity in patients with *C. acnes* considered a contaminant was more prolonged than in patients with *C. acnes* VO.

Treatment of *C. acnes* VO is non-standardized. While surgery may be warranted in patients with hardware-associated infection, most patients with *C. acnes* VO can likely be treated with antimicrobial therapy alone (Berbari et al., 2015). In the case series mentioned above, six of nine patients were treated surgically. In the current series, 14 of 16 patients were successfully treated with antimicrobial therapy alone. *C. acnes* is generally susceptible to β -lactams, including third-generation cephalosporins, fluoroquinolones, rifampin, and doxycycline, albeit resistant to metronidazole. Increasing resistance to clindamycin is reported, likely due to its extensive use in treating acne vulgaris (Boisrenoult, 2018; Mercieca et al., 2020). The most used parenteral antibiotics in this study were β -lactams, particularly ceftriaxone. Oral therapy is a viable alternative for patients with uncomplicated VO (Spellberg et al., 2022). In this cohort, three patients were successfully treated with oral therapy alone (two with moxifloxacin and one with doxycycline). Since some microbiology laboratories do not routinely report susceptibility to all antibiotics potentially considered for therapy, it may be beneficial to obtain that testing upfront, even if testing needs to be performed in a reference laboratory.

A strength of this study is the relatively large size of the cohort and the use of a strict case definition of *C. acnes* VO. This typically represents a challenge because it can be difficult to distinguish *C. acnes* as a true pathogen versus a clinically insignificant isolate. The best approach between treatment, watchful waiting, or another diagnostic procedure depends on the clinical scenario. A reasonable approach is proposed, according to the findings of the study. In cases of monomicrobial infection, if there is a strong suspicion of VO from the patient's clinical and radiological characteristics and risk factors (such as a previous intervention in the spine), it is reasonable to initiate treatment, even without a second confirmatory culture. In contrast, if suspicion of clinical infection is low, a repeat biopsy or close follow-up with repeat imaging 4–6 weeks later may be considered. If there is no suspicion for VO, as in cases of biopsy performed for malignancy, a wait-and-see approach is preferred. Another strength is that data are provided on the long-term follow-up of patients with suspected contamination with *C. acnes* who were not treated. Most previous studies focus on confirmed or suspected infections. According to the results of this study, in the absence of suspicion of VO, isolation of *C. acnes* does not require treatment. Larger data sets are warranted to confirm this result.

This study has limitations. First, it was a retrospective analysis, and some information may need to be included. For example, five of 16 patients with VO did not have a histopathological examination of the tissue sample. Histopathological analysis constitutes a valuable additional tool for diagnosing VO, and differentiating between true pathogen and contaminant and is recommended (Weihe et al., 2022). Furthermore, some patients had just one sample submitted for culture. In our case series, 13/16 in the VO group and 8/21 in the contaminant group had ≥2 cultures performed. Specifically, 6/13 patients in the VO group had a single culture growing *C. acnes*. It is recommended to submit more than one sample in the setting of suspected osteoarticular infection, since it can be challenging to interpret the results of single positive cultures. Also, since there is no established definition for *C. acnes* VO, the results and approach may be different from others. Moreover, laboratory techniques have changed over the last 10 years and may have affected the culture-based findings.

In conclusion, the clinician should consider *C. acnes* as an agent of VO, especially if a previous spinal procedure was performed. Treatment with parenteral β -lactams appears to be effective, but targeted oral therapy could be a valid alternative. Spine cultures should be anaerobically incubated for 14 d. In the absence of clinical and radiological signs of VO, it is reasonable to consider the isolation of *C. acnes* from a single culture as clinically insignificant. Given the rarity of *C. acnes* VO, the conclusions made here are based on a small number of subjects. A multicenter study or a systematic review comprising a more significant number of patients is needed to confirm the results presented.

## Data Availability

Data are available upon request.
